# Genetic differentiation over a small spatial scale of the sand fly *Lutzomyia vexator* (Diptera: Psychodidae)

**DOI:** 10.1186/s13071-016-1826-5

**Published:** 2016-10-18

**Authors:** Allison T. Neal, Max S. Ross, Jos J. Schall, Anne M. Vardo-Zalik

**Affiliations:** 1 Department of Biology, Norwich University, Northfield, VT 05663 USA; 2 Department of Biology, University of Vermont, Burlington, VT 05401 USA; 3 Department of Biology, Pennsylvania State University, York, PA 17403 USA

**Keywords:** Sand flies, *Lutzomyia*, Genetic differentiation, Microsatellites

## Abstract

**Background:**

The geographic scale and degree of genetic differentiation for arthropod vectors that transmit parasites play an important role in the distribution, prevalence and coevolution of pathogens of human and wildlife significance. We determined the genetic diversity and population structure of the sand fly *Lutzomyia vexator* over spatial scales from 0.56 to 3.79 km at a study region in northern California. The study was provoked by observations of differentiation at fine spatial scales of a lizard malaria parasite vectored by *Lu. vexator*.

**Methods:**

A microsatellite enrichment/next-generation sequencing protocol was used to identify variable microsatellite loci within the genome of *Lu. vexator*. Alleles present at these loci were examined in four populations of *Lu. vexator* in Hopland, CA. Population differentiation was assessed using Fst and D (of Cavalli-Sforza and Edwards), and the program Structure was used to determine the degree of subdivision present. The effective population size for the sand fly populations was also calculated.

**Results:**

Eight microsatellite markers were characterized and revealed high genetic diversity (uHe = 0.79–0.92, Na = 12–24) and slight but significant differentiation across the fine spatial scale examined (average pairwise *D* = 0.327; *F*_*ST*_ = 0.0185 (95 % bootstrapped CI: 0.0102–0.0264). Even though the insects are difficult to capture using standard methods, the estimated population size was thousands per local site.

**Conclusions:**

The results argue that *Lu. vexator* at the study sites are abundant and not highly mobile, which may influence the overall transmission dynamics of the lizard malaria parasite, *Plasmodium mexicanum,* and other parasites transmitted by this species.

**Electronic supplementary material:**

The online version of this article (doi:10.1186/s13071-016-1826-5) contains supplementary material, which is available to authorized users.

## Background

Sand flies (*Lutzomyia*) are important vectors of *Leishmania* and other cellular and viral pathogens of medical, veterinary and wildlife significance throughout the Americas [1, 2]. Unraveling the ecology and genetic structure of these insects over a geographical landscape is required for understanding epidemiological patterns, for public/veterinary health efforts in vector control and for understanding wildlife disease dynamics. For example, because sand flies are relatively weak fliers [3], their dispersal capability may be limited, resulting in a structured population over a small spatial scale, leading to localized transmission of disease. Additionally, vectors with limited dispersal could locally adapt with specific pathogen genotypes, altering not only vector competence but also disease manifestations [4]. This is the picture predicted by the “geographic mosaic model of coevolution” of Thompson [5] which posits that founder effect, genetic drift and selection superimposed over a geographic landscape will result in extremely complex patterns for interacting species such as parasites and their hosts.

Previous surveys examining *Lutzomyia* population structure used both biochemical and DNA markers and have yielded conflicting results. While some studies show significant genetic structure over rather small spatial scales, others suggest a panmictic breeding structure for the most important sand fly vectors of *Leishmania* (reviewed in [4]). Because the majority of research on *Lutzomyia* genetics has focused on medically important vectors (including *Lu. longipalis*, *Lu. peruensis*, *Lu. ayacuchensis* and *Lu. shannoni* [4, 6–9]), we know very little in regards to *Lutzomyia* spp. that transmit wildlife diseases and how the structure of these vector populations may influence disease patterns. Examining these wildlife disease vectors will not only provide insights as to how diseases are maintained in natural settings, but these systems can also serve as natural models for medically important vectors that may be difficult to study.

In North America, two species of *Lutzomyia*, *Lu. vexator* and *Lu. stewarti*, are the vectors for the lizard malaria parasite, *Plasmodium mexicanum*, a parasite of the western fence lizard *Sceloporus occidentalis* in northern California, USA [10]. *Plasmodium mexicanum* is the only *Plasmodium* species known not to be vectored by a mosquito; otherwise, the life-cycle concords with that of other *Plasmodium* species, and molecular phylogenetic studies show it falls within the overall *Plasmodium* clade [11]. This parasite-host system has been under study at a site in California for almost four decades [12], yet we still know relatively little regarding the biology and genetics of the vectors. Several patterns have emerged from previous studies that suggest the California sand flies are distributed in an ecological and genetic mosaic over small spatial scales. First, the prevalence of the parasite in the lizard host varies among sites that are only hundreds of meters apart, and this variation has held for decades [13, 14]. That is, there are nearby sites with consistently low versus high relative prevalence over many years. This suggests that the transmission ecology consistently varies among sites over short distances. Second, although infection prevalence in the lizard can reach 35 % at some sites, the sand flies are often difficult to find and collect at those sites [13]. This begs the question of the population density of the vectors. Third, a study using microsatellite genetic markers found that the parasite differs genetically among sites < 1 km apart, but the lizard appears panmictic among sites > 40 km distant [14]. This would be explained if infected lizards remain local whereas related, but not infected, lizards move readily, which would drive gene flow of the vertebrate host but leave the parasite behind at local sites. Supporting this hypothesis, infected fence lizards experience physiological and behavioral deficits, including a reduction in activity in their home range [14, 15]. However, for such small-scale genetic structure to be preserved in the parasite, it must also not be moved by its vector, suggesting the potential for low migration rates in the sand fly.

Although we were primarily interested in determining the local structure of the most common insect host of *P. mexicanum*, *Lu. vexator*, as it relates to patterns of malaria prevalence, this research also has larger-scale applications. *Lutzomyia vexator* is widespread across the United States [16], and may be an important vector for other wildlife diseases [17]. Additionally, the proximity of *Lu. vexator* sand flies to kennels where an *L. infantum* outbreak occurred in fox hounds presents the question as to whether or not *Lu. vexator* could potentially vector mammalian *Leishmania* [18]. Taken together, understanding the genetic structure of this important sand fly vector could help researchers address a multitude of questions concerning *Lutzomyia* sand flies in the United States and their potential to serve as disease vectors.

To better understand these issues, we surveyed the genetic diversity of *Lu. vexator* at several sites using microsatellite genetic markers. Our goals were first, to assess any population structure of the sand flies over sites from ~0.5 to ~3.8 km apart. The genetic differentiation of the parasite, but not lizard, suggests that the sand flies are not very mobile among sites and would thus be differentiated among local patches of the habitat. This could create an environment favorable for local adaptation of parasite/vector genotypes, with populations varying in their susceptibility to environmental disturbances. Secondly, to estimate the effective (breeding) population size of *Lu. vexator*. We predict that the actual population size is much greater than what is detected by standard trapping methods. Thirdly, to provide results of our scanning of the sand fly’s genome for microsatellite regions that may be useful for other researchers.

## Methods

### Study sites

**Fig. 1:**
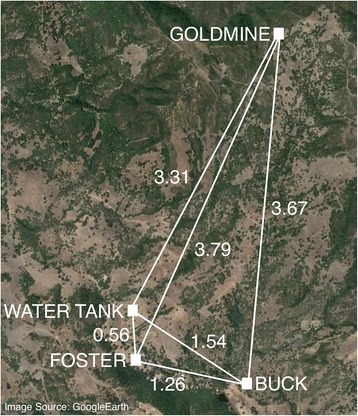
Sand fly (*Lutzomyia vexator*) sampling locations at the University of California Hopland Research and Extension Center. Distances between sites are given in kilometers. Map data: Google

We conducted the study at the University of California Hopland Research and Extension Center (HREC), a 2168 ha property near the town of Hopland, California, USA. This site has been the focus of a long-term lizard malaria study since 1978 [12, 19]. The habitat is topographically complex, ranging from 152 to 914 m elevation, with a Mediterranean climate of hot, dry summers from June to September and rainy mild winters, with 75 % of the annual rainfall occurring between November and February. The frost-free growing season lasts approximately 250 days (HREC weather data). Four sites were chosen for collecting sand flies based upon the presence of active ground squirrel (*Otospermophilus beecheyi*) colonies; sand flies use ground squirrel burrows as day-time resting sites and females deposit eggs in the organic-rich feces [20]. The sand flies from these sites are considered here as separate populations for analysis; Fig. 1 shows the location of these sites relative to one another with the GPS coordinates provided in Additional file 1: Table S1. The majority of burrows at each site were trapped, but due to burrow location, opening size and angle, not every burrow could be sampled. The characterization of each site is as follows: Buck: grassy terrain with scattered oak and madrone trees. The majority of the rodent burrows trapped were on the gentle slope of a grassy, exposed hill with an elevation of approximately 300 m. The ground squirrel colony consisted of at least 40 burrows. Water Tank: a relatively flat site with grassy terrain, no tree cover and an elevation of approximately 240 m. The ground squirrel colony consisted of approximately 20 burrows. Foster: a tree-covered bank off the side of an access road, covered in leaf litter with an approximate elevation of 260 m. The ground squirrel colony was small, with approximately 10 burrows. Goldmine: grassy terrain with scattered trees and downed logs, with an approximate elevation of 850 m. The ground squirrel colony consisted of at least 40 burrows.

### Sand fly collection

On 24 nights from 8 July 2012 to 3 August 2012, sand flies were collected by funnel traps set over entrances of ground squirrel burrows in the late afternoon, and flies were collected from 20:00 to 24:00. Two to three sites were trapped each night, with each site trapped a total of 10–18 nights. Species identification was by the criteria of Young and Perkins [21]. *Lutzomyia vexator* was more common (~89 % of sand flies) than *Lu. stewarti*, so only *Lu. vexator* was used for the study. After identification, flies were stored individually in vials containing 100 % ethanol.

### Microsatellite survey

To date, microsatellite markers have only been characterized for two species of *Lutzomyia* sand fly, *Lu. whitmani* [22] and *Lu. longipaplis* [23], both vectors for human leishmaniasis. We initially tested a panel of microsatellite loci for *Lu. longipalpis* [23], but these did not work for *Lu. vexator*. Therefore, we identified new microsatellite loci for *Lu. vexator* to be used in this study. Six *Lu. vexator* individuals were pooled and DNA was extracted using the Qiagen DNeasy kit, following the supplier’s protocol (Qiagen, Valencia, USA). DNA quantity and quality were assessed with a Nanodrop spectrophotometer (ThermoFisher Scientific, Wilmington, USA), and DNA was submitted to the Cornell University Evolutionary Genetics Core Facility (Ithaca, New York). There, using the method of Hamilton et al. [24], genomic DNA was Hinc H digested and enriched for microsatellites using a panel of 14 biotinylated probes. Fragments were then sequenced using the Titanium 454 platform (454 Life Sciences, Branford, USA).

Our goal was to identify microsatellite loci that were variable and that consistently amplified for *Lu. vexator*. First, sequences (FASTA files) containing a microsatellite were examined to select those with longer repeats, which are more likely to be variable [25]. Sequences that were represented multiple times with identical flanking regions, but different number of repeats, were also selected, as these sequences showed variation. After choosing loci for further testing, PCR was performed on DNA extracted from individual sand flies. Loci that produced a clear band on an agarose gel were selected for further testing by PCR using fluorescently-labeled forward primers. Loci that produced clear results and variation after running the PCR product on a 3730*xl* Genetic Analyzer (ThermoFisher Scientific, Wilmington, USA) were selected for our final panel of markers. Eight loci were chosen for use in this study (Table 1; GenBank accession numbers KT693036–KT693043) and thermal cycler programs were optimized to provide consistent results (below).

**Table 1:** The eight microsatellite loci used to examine genetic diversity in a sand fly *Lutzomyia vexator* at a site in northern California, the University of California Hopland Research and Extension Center. For each locus, the GenBank accession numbers, motif, length (in nucleotides) of expected amplicon at given repeat length and PCR primers are presented

Locus	GenBank	Motif	Length	Primers (5′–3′)
Lvx9	KT693036	AG	172 (19×)	F: CCGAATTGTCGAACGATTTG
				R: GTCAATTTGATGCTCTCGTAC
Lvx67	KT693037	AG	165 (18×)	F: CCAAGAATCGCATATCAACATG
				R: GCTTCATCCTGTATTCATGG
Lvx123	KT693038	TC	318 (22×)	F: CCTTATTCTCACTTGCATCTCGG
				R: AGAGAAGATAGAGCTCCATTGGG
Lvx179	KT693039	CT	296 (22×)	F: CGCAAACATGCTGATAAAGAATGC
				R: GGACTTTGTTGCATTGCAGC
Lvx7442	KT693040	AG	323 (19×)	F: GCTTCCAAAGAGGAAGGTGAG
				R: GGATACACTCGAAAATTGGTGC
Lvx504	KT693041	TC	403 (25×)	F: GTTCTTTAAGACGCGTGAAATGC
				R: AAATTCTCATTGGGCAGGATAGC
Lvx90606	KT693042	CTT	246 (12×)	F: GTCTCAATACGCGTCTCTCAAAG
				R: GTTAGTGCGAGAGGCAGAGTC
Lvx918	KT693043	AG	301 (15×)	F: GGTTGAACAACAGTCAGCTAAG
				R: GCCTAAAGGAAACGTAGCATTCTC

*Abbreviations*: *F* forward, *R* reverse

### Fly genotyping

Each sand fly was removed from alcohol and air dried. The head/thorax of each fly was removed and used for genetic analysis. The abdomen was not included to prevent including genotypes from potential mates (stored sperm). DNA from each fly was extracted using the DNeasy kit (as above). For the eight loci, PCR was run using Ready-to-Go beads (GE Healthcare, Piscataway, USA) that contained DNA polymerase, dNTPs and buffers. A 25 μl reaction included one bead, 21 μl water, 1 μl of each 10 μM forward and reverse primer and 2 μl of the extracted DNA. The forward primer was fluorescently labeled with 6FAM dye (Integrated DNA Technologies, Coralville, USA). A negative control was included for each group of samples processed. The PCR conditions were as follows: initial denaturation at 94 °C for 3 min, followed by 33 cycles of 94 °C/50 s, 51 °C/30 s and 72 °C/2 min, with a final extension at 72 °C for 15 min.

PCR products were run on a 1 % agarose gel and the density of the band used to determine the dilution of the samples for genotyping. The diluted product (1 μl) was added to a 15 μl mix of LIZ500 size standard and Hi-Di formamide (Applied Biosystems by Life Technologies, Foster City, USA). These samples were then processed through a 3730*xl* Genetic Analyzer (as above), and data was visualized as pherogram graphs using PeakScanner 1.0 software (Applied Biosystems). The pherograms were inspected to score each sand fly’s genotype by allele size (in base pairs).

### Analysis

Before analysis, the data were checked for statistical evidence of scoring errors, large allele dropout or null alleles using the program MICRO-CHECKER [26]. Summary statistics for each locus were calculated using GenAlEx version 6.501 [27] and included number of detected alleles (Na), effective number of alleles (Nea; the number of equally frequent alleles that would give the same heterozygosity in an ideal population), unbiased estimate of expected heterozygosity (uHe; a measure of genetic diversity) and observed heterozygosity (Ho; proportion of sand flies heterozygous at that locus). A test for deviation from expected Hardy-Weinberg equilibrium proportions by a Markov-chain method was performed using GENEPOP online version 4.2 [28, 29]. Default values for dememorization number and number of iterations were used and the number of batches was increased to 2000 to ensure that the resulting standard errors were all less than 0.01. Bonferonni correction was used to adjust the cutoff for statistical significance due to the large number of comparisons being made.

The *Lutzomyia* haploid number of chromosomes is only four [30], so linkage disequilibrium (LD) is likely even when using a small number of markers, especially if they are closely positioned on a chromosome or if there is substantial inbreeding at a site. The Markov chain method implemented in GENEPOP was used to test for deviations from linkage equilibrium. Default values were used for dememorization number and number of iterations. The number of batches was increased to 3000 so that the resulting standard errors were less than 0.01. The cutoff for statistical significance was again adjusted using the Bonferonni method.

Because we found evidence of null alleles at one of the loci (see Results), we opted to use the program FreeNA [31] to estimate genetic differentiation between sites. This program calculates standard measures of population differentiation (*F*_ST_) and genetic distance after correcting for null alleles. Correction involves estimating the frequency of null alleles and assigning them an allele number not previously present in the data set [31]. Both *F*_ST_ and D are useful for understanding population genetic patterns. Genetic distance, here reported as the genetic distance of Cavalli-Sforza and Edwards (D) [32], reports how different two populations are genetically and can be used to compare between populations. These values increase as genetic differences between populations become greater. *F*_ST_ ranges from zero, for complete panmixia between two sites, to one, for complete genetic differentiation (all private alleles). Values of *F*_ST_ that are significantly different from zero suggest population substructuring, with some limitation of migration between local subpopulations. To verify the analysis in FreeNA, we also performed a test for allelic differentiation using Fisher’s exact test implemented in GENEPOP online version 4.2 [28, 29]. Results qualitatively matched those obtained from FreeNA and are not shown.

In addition to calculating standard measures of population differentiation for our four populations, we wanted to determine whether any differences among populations were strong enough to be identified by clustering algorithms. We therefore used STRUCTURE Version 2.3.4 [33] to determine how many genetic clusters were present in our data and whether these clusters corresponded to the flies’ capture site. This program assumes that populations are in Hardy-Weinberg and linkage equilibrium and uses Bayesian methods to group individuals into a user-defined number of clusters (K) that minimizes Hardy-Weinberg and linkage disequilibrium. We ran this analysis using an admixture model with independent allele frequencies with a burn-in of 50,000 followed by 100,000 iterations of the Markov Chain Monte Carlo for K from one to seven. All analyses were run both without prior information regarding capture site and with prior information about capture site. The STRUCTURE documentation indicates that including prior information about capture locations can aid in population assignment for difficult datasets [33], which likely applies here given the small spatial scale the samples were taken from. Data from locus Lvx90606 was excluded due to the presence of null alleles (see Results). To determine the best K, we calculated the posterior probability for each K using the equation provided in the STRUCTURE documentation and selected the K with the highest value [33].

Finally, we wanted to obtain a rough estimate of the population size of these flies. Many methods have been proposed to estimate breeding population size (the effective population size, Ne), all with shortcomings [34]. The general assumption underlying estimates is that a greater genetic diversity reflects a larger breeding population and thus Ne can be estimated if genetic diversity and mutation rate are known [35]. We were interested in a measure of absolute Ne; that is, we wanted to know if the Ne was large, despite our inability to collect large numbers of flies. Measures of relative population size are useful to compare among sites [36], but not relevant here. Therefore, we use the estimate of genetic diversity and microsatellite mutation rates and the equation *N*_e_μ = 1/8 {[1/(1 − *H*)]^2^ − 1}. We assumed a step-wise mutation model and used a mutation rate of 10^-4^ [37].

## Results

### Sand fly collections

Over the 24-night collection period, 213 sand flies were collected (190 *Lu. vexator*, 13 *Lu. stewarti* and ten unidentified) across the four different sites; ‘Buck’ was the most prolific site, with 111 flies collected. As the numbers of flies collected from each of the other sites were substantially lower, we only used 62 flies from Buck for analysis. Our analysis utilized 152 *Lu. vexator* flies in total (sample sizes: buck = 62; water tank = 38; foster = 18; goldmine = 34).

### Recovered microsatellites

The enrichment and sequencing protocol produced 2279 sequences (assembled contiguous sequences, or contigs) containing a microsatellite repeat. The complete list of the contigs with repeat motif, suggested PCR primers and annealing temperatures and the FASTA files are provided in Additional files 2 and 3: Table S2 and Dataset S1, respectively. The most common repeat motifs were two base repeats (total = 1712), three base repeats (256) and four base repeats (294). These repeat motifs, of course, depended in part on the probes used during the enrichment protocol.

### Summary statistics on allelic variation

Over all sites, 152 sand flies were included in the study, with 144–152 successfully genotyped per locus. Failure to amplify a sand fly for any locus could be a result of null alleles, but only one locus (Lvx90606) showed evidence of null alleles. A large number of alleles were detected for most loci (11–24, with 4/8 loci having at least 20 alleles; Table 2). The effective number of alleles, however, was smaller (4.7–11.8, Table 2).

**Table 2:** Summary statistics on variation at eight microsatellite loci for the sand fly *Lutzomyia vexator* collected at the University of California Hopland Research and Extension Center site in northern California. If the locus displayed a significant deviation from Hardy-Weinberg equilibrium (*P* < 0.00625, Bonferonni correction), the origin is given as a surplus of heterozygotes (+) or a deficit (−)

Locus	*n*	Na	Nea	uHe	Ho	*P*
Lvx9	2	12	4.9	0.796	0.793	0.3648
Lvx67	4	20	9.6	0.899	0.872	0.0561
Lvx123	4	18	4.7	0.788	0.797	0.1879
Lvx179	2	24	7.9	0.876	0.847	0.0238
Lvx7442	0	23	5.7	0.827	0.895	0.0006 (+)
Lvx504	1	22	11.8	0.918	0.874	0.0134
Lvx90606	8	12	6.2	0.842	0.590	< 0.0001 (−)
Lvx918	0	19	9.7	0.900	0.914	0.0412

*Abbreviations*: *n* the number of sand flies that did not amplify of the 152 sampled, *Na* number of observed alleles, *Nea* effective number of alleles, *uHe* unbiased estimate of heterozygosity, *Ho* observed heterozygosity, *P* significance for deviation from Hardy-Weinberg equilibrium by Markov chain

When all populations were combined, deviation from Hardy-Weinberg equilibrium (HWE) was observed for two of the eight loci (*P* < 0.00625; Table 2) and linkage disequilibrium (LD) was detected for six out of 28 pairs of loci (*P* < 0.00178; Additional file 4: Table S3). Because both deviations from HWE and LD can result from differentiation among sites, data were parsed by site. For one of the loci with significant deviations from HWE, the deviations disappeared when the data were parsed by site. The other locus (Lvx90606) showed evidence of null alleles (see above) and deviated from HWE at two of the four sites and was bordering on significance at the other two (buck: *P* < 0.0001; foster: *P* = 0.0056; goldmine: *P* = 0.0023; water tank *P* < 0.0001). Similarly, when data was parsed by site, only 5/112 site and locus combinations showed significant linkage disequilibrium, and three of these significant deviations were between Lvx 504 and Lvx7442 (buck: *P* < 0.0001; foster: *P* < 0.0001; water tank: *P* < 0.0001). The other two were other combinations of loci at Buck (123 & 504: *P* < 0.0001; 918 & 7442: *P* < 0.0001).

### Differentiation among sites and effective population size

**Fig. 2:**
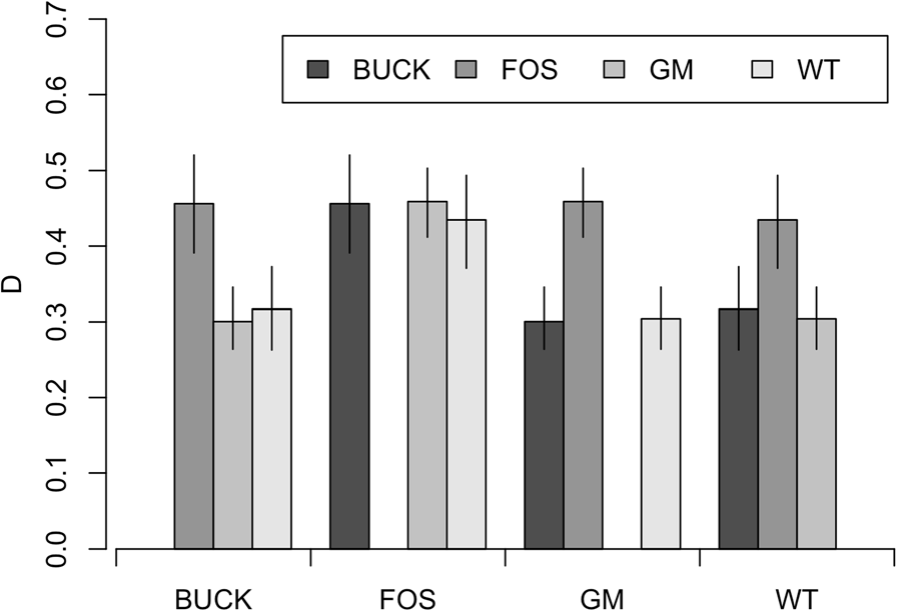
Pairwise genetic distance (D) estimates for sand flies (*Lutzomyia vexator*) collected from four sites at the University of California Hopland research and extension center. Error bars show a 95 % confidence interval obtained by bootstrap resampling. *Abbreviations*: BUCK, buck; FOS, foster; GM, goldmine; WT, water tank

Population differentiation across all sites and loci using the correction for null alleles showed signs of differentiation (*F*_ST_ = 0.0185; 95 % bootstrapped CI: 0.0102–0.0264); *F*_ST_ for individual pairs of sites ranged from 0.00945 to 0.0376 (Table 3). None of the 95 % CI included zero, suggesting significant, if slight, genetic differentiation among all sites sampled. The average genetic distance (D) among sites was 0.379. The 95 % CI for each site suggest that flies from Foster are more different from the other sites than the other sites are from one another (Fig. 2).

**Table 3:** Site-by-site *F*_ST_ values comparing *Lutzomyia vexator* collected from four sites at the Hopland Research and Extension Center in Hopland California. Values above the diagonal are point estimates of *F*_ST_, while values below the diagonal are 95 % bootstrapped confidence intervals

Site	Buck	Foster	Goldmine	Water tank
Buck	–	0.038	0.0099	0.014
Foster	0.021–0.053	–	0.036	0.029
Goldmine	0.0040–0.017	0.022–0.049	–	0.0095
Water Tank	0.0051–0.026	0.015–0.042	0.0017–0.021	–

**Fig. 3:**
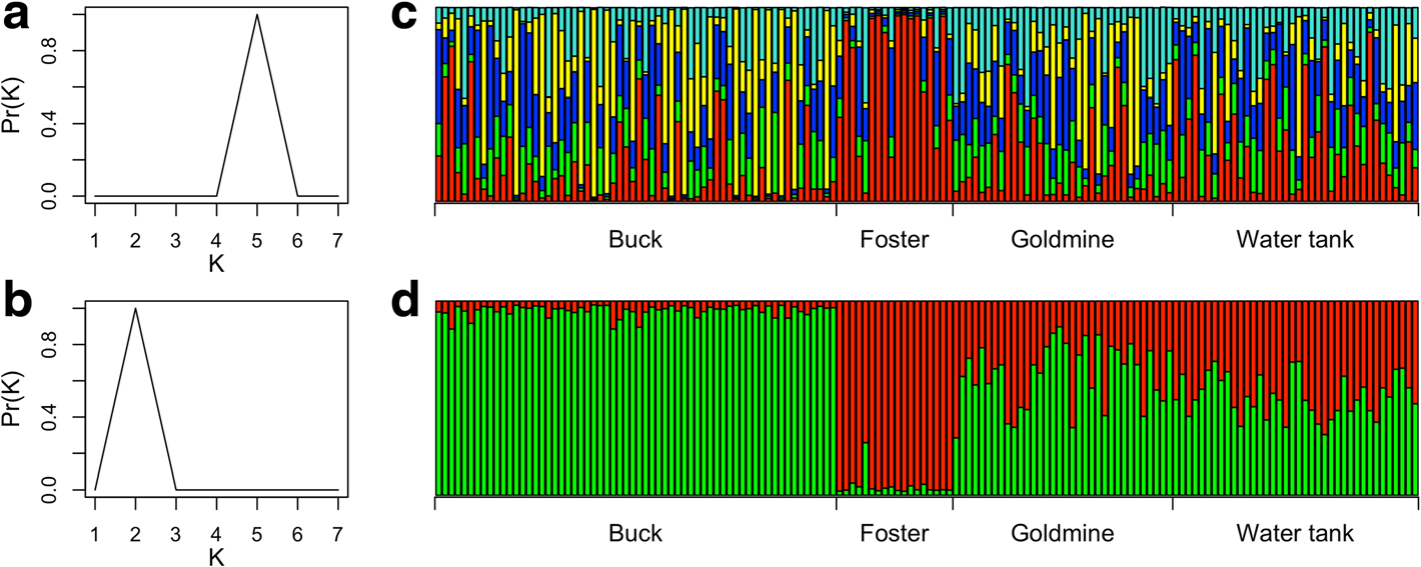
Results from clustering analysis using the genotypes of sand flies (*Lutzomyia vexator*) captured at four sites at the University of California Hopland Research and Extension Center. Panels **a** and **b** show estimated posterior probabilities of each value of K. Panels **c** and **d** show the proportional assignment of each individual (each vertical bar) to one of the clusters. Panels **a** and **c** show results when no prior location information is supplied. Panels **b** and **d** show results when prior location information is supplied

Clustering analysis showed that at least some of this genetic differentiation is strong enough to create detectable genetic clusters within the data. Analysis with no prior capture location information indicates five genetic clusters, while analysis with prior location information indicates two distinct clusters (Fig. 3a, b). The two clusters identified by STRUCTURE using prior location information roughly correspond to flies from Foster and flies from Buck, with flies from Goldmine and Water Tank appearing to have genotypes intermediate between the two (Fig. 3d). The five clusters identified by STRUCTURE without prior location information are less well defined, although most of the flies from Foster again appear to group well together (Fig. 3c).

Genetic diversity varied both among sites and among loci (Table 2), so estimates of Ne also varied: Buck Ne [range for all loci] = 29–132 × 10^3^; Foster Ne = 10–67 × 10^3^; Goldmine Ne = 28–237 × 10^3^; Water tank Ne = 22–118 × 10^3^. Thus, by all estimates, the breeding population size was in the order of tens of thousands at each site.

## Discussion

Our study on the population structure of the sand fly *Lutzomyia vexator* at a study region in northern California was prompted by two observations on a lizard malaria parasite, *P. mexicanum*, which is vectored by *Lu. vexator*. First, the parasite is genetically differentiated over a fine spatial scale, which suggests the insect may also have a viscous population structure, even at spatial scales over a few hundred meters. Second, the high prevalence of *P. mexicanum* in its lizard host would presumably require a large population size for the sand fly despite the difficulty in collecting the insects using standard methods. The study also has broader implications because of the importance of *Lutzomyia* sand flies for human public health.

Using a panel of eight microsatellite loci, we found exceptionally high genetic diversity in the *Lu. vexator* population, with heterozygosity estimates > 0.75 for each locus. Another measure of genetic diversity, the number of alleles per locus, was also high for *L. vexator* (12–24 alleles/locus). While we did select for microsatellite loci that were variable, such high levels of genetic diversity are not uncommon and have been reported for other sand fly taxa [23, 38–40].

Both measures of genetic structure (*F*_ST_ and D) indicated genetic differentiation between sites that were approximately 0.5–3.8 km distant, and the STRUCTURE analysis provides further evidence that there are at least two genetically distinct sand fly populations at our study site. Previous studies that have documented significant levels of population differentiation for *Lutzomyia* sand flies did so over larger collection distances (e.g. *L. longipalpis* populations in Brazil) [6, 41]. The degree of differentiation in our study was slight, but still remarkable for such short distances for a flying insect that can be carried by wind currents. The sand flies, though, do not emerge from their burrows even under slight wind movement [19], which could account for their local structure. Additionally, researchers estimate that most sand flies do not travel much more than one kilometer throughout their lifetime, which could account for the pattern we observed [42]. Because *Lu. vexator* is often found in ground squirrel burrows, which are patchily distributed across the research center, the effect of limited dispersal may be amplified in this system, leading to detectable genetic differentiation even on a small spatial scale.

The STRUCTURE analysis (Fig. 3) and comparisons of D for different pairs of populations (Fig. 2) also suggest that flies from Foster are the most genetically distinct when compared with other flies despite the relatively central location of this site (Fig. 1). We note that of the four sites, the Foster site is the most densely treed and the closest to the road and field station buildings. This site also had the fewest active ground squirrel burrows, which may suggest that the ground squirrel and sand fly populations in this region of the study site may not yet be well established. The fact that this site is centrally located and has a different allelic make-up (as indicated by the *F*_ST_ and STRUCTURE analyses) than the other sites suggests that differences in microhabitat rather than geographic distance drive some of the small-scale patterns in the genetics, as has been suggested for at least one other sand fly species (i.e. *Phlebotomus papatasi*) [41].

A potential source of error in the data is our unbalanced sample sizes among sites. Generally, equal population samples are preferred, especially for *F*_ST_ analysis, and the small sample size at Foster (18 flies) may seem particularly troubling to some. Our intuition suggested that this small sample size would be more likely to lead to type II errors (i.e. being unable to detect true differentiation between populations) rather than type I (i.e. detecting false signals of differentiation), and a simple simulation using randomized data that preserved allele frequencies (over all populations) and site sample sizes confirmed that the differences in sample size among sites alone does not create false patterns of population differentiation (data not shown). We therefore have reason to believe that the patterns observed in our data are real, or at least are not generated by differences in sample size among sites.

The results of this study may also shed some light on the relative abundance of sand flies in this area. Over the past three decades, the lizard malaria parasite *P. mexicanum* has been studied in its lizard host and some years the overall prevalence in the lizards can reach > 35 %. Laboratory studies show that > 90 % of sand flies do not survive past laying a clutch of eggs [20], which would most likely require a very large vector population to maintain the parasite at such a high prevalence level. After 24 nights, setting up > 40 traps per night, only 190 *Lu. vexator* flies were collected. Likewise, other researchers have reported low collection yields for sand flies after intensive efforts (e.g. *Lu. vexator* was found in New York State, but only a few sand flies were captured [18]). Using a simple model relating microsatellite mutation rate and genetic diversity to the breeding population size, we estimated the effective population size of *Lu. vexator* at tens of thousands per capture site. Even if this model deviates from the actual effective population size by a factor of ten, the population size would be very dense. The high genetic diversity and estimated effective population size of the sand flies at our sites argues that the insects are common, yet cryptic to investigators. This result suggests that the sand flies may be laying eggs in locations other than the rodent burrows. The use of modified CDC light traps (with CO_2_) or modified Sherman traps/emergence traps to collect from tree holes/leaf litter could capture additional sand flies from these sites [16, 43–45].

The intent of our study was to examine the genetic structure of a sand fly species over a small spatial scale to determine if vector population subdivision could contribute to the patchy distribution of malaria we observe at our field sites. While our results indicate that the *Lu. vexator* population is structured, it is not yet clear the extent to which this structure is important biologically and whether it might result in localized transmission of malaria at the field site. However, the fact that we have some structure at this spatial scale raises questions concerning the implications for this widespread species. If even slight genetic differentiation occurs on such a small scale, it is possible that *Lu. vexator* populations across North America are not one species and cryptic species may exist. Examining behavioral, biochemical and morphological differences in populations of *Lu. vexator* may reveal otherwise hidden diversity. Questions are also raised about the possibility of local adaptation, such as whether the presumed limited dispersal for *Lu. vexator* may allow for local adaptation between vector and parasite. Such patterns have been documented previously for other *Plasmodium-*host associations [46, 47]. While the level of differentiation documented in our study may be too minimal to allow for adaptation on such a small spatial scale, local adaptation may occur over larger scales. Further studies examining oocyst burden and vector competence for different *P. mexicanum*/*Lu. vexator* population pairings could determine the effects of this differentiation on the transmission biology of this parasite. The genetic structure of this disease vector can serve as a model system to understand sand fly/pathogen transmission dynamics across small and large spatial scales.

## Conclusions

We present evidence that the population of *Lu. vexator* in Hopland, CA is structured, with at least two genetically differentiated populations existing within 0.5–3.8 km of each other. This structuring suggests reduced dispersal of sand flies across the field site and may contribute to the observed patchy distribution of malaria. Our findings emphasize that disease vectors can be differentiated over small spatial scales and further studies should be conducted to assess the impacts of such differentiation on disease transmission.

## Additional files

Additional file 1: Table S1. Geographic coordinates of four sites used in the study of genetic differentiation of a sand fly, *Lutzomyia vexator*, in northern California, USA. (DOC 27 kb)
Additional file 2: Table S2. Motifs and PCR primer suggestions for microsatellite loci recovered from the enrichment protocol. Abbreviations: Tm, recommended annealing temperature. (XLSX 170 kb)
Additional file 3: Dataset S1. FASTA files for sequences recovered during search for microsatellite markers in the genome of *Lutzomyia vexator*. Contig number, the number of times the Contig was recovered, the length of the Contig and sequence are given. (TXT 44429 kb)
Additional file 4: Table S3. Results from a statistical test for linkage disequilibrium for eight *Lutzomyia vexator* loci. * indicates *P* < 0.0018 (0.05/28, Bonferonni correction); ns indicates *P* > 0.0018. (DOCX 13 kb)

## Abbreviations

CDC: Center for Disease Control and Prevention
D: Genetic distance of Cavalli-Sforza and Edwards
Ho: Observed heterozygosity
HREC: University of California Hopland Research and Extension Center
LD: Linkage disequilibrium
*n*: number of samples that did not amplify for a particular locus
Na: Number of detected alleles
Nea: Effective number of alleles
uHe: unbiased estimate of expected heterozygosity
