# Supplementary material for: Genetic differentiation over a small spatial scale of the sand fly Lutzomyia vexator (Diptera: Psychodidae)
Source: Parasit Vectors. 2016 Oct 18;9:550. doi: 10.1186/s13071-016-1826-5 (PMC5070220; doi:10.1186/s13071-016-1826-5)
Supplement: Additional file 1: Table S1. — Geographic coordinates of four sites used in the study of genetic differentiation of a sand fly, Lutzomyia vexator, in northern California, USA. (DOC 27 kb) [file 13071_2016_1826_MOESM1_ESM.doc]

Additional File 1: Table S1. Geographic coordinates of four sites used in the study of genetic differentiation of a sand fly, *Lutzomyia vexator*, in northern California, USA.

| Goldmine | 39° 1'56.59"N | 123° 4'9.71"W |
| --- | --- | --- |
| Water Tank | 39° 0'22.12"N | 123° 5'14.78"W |
| Foster | 39° 0'4.07"N | 123° 5'10.91"W |
| Buck | 38°59'57.55"N | 123° 4'19.69"W |
