# Supplementary material for: Genetic differentiation over a small spatial scale of the sand fly Lutzomyia vexator (Diptera: Psychodidae)
Source: Parasit Vectors. 2016 Oct 18;9:550. doi: 10.1186/s13071-016-1826-5 (PMC5070220; doi:10.1186/s13071-016-1826-5)
Supplement: Additional file 4: Table S3. — Results from a statistical test for linkage disequilibrium for eight Lutzomyia vexator loci. * indicates P < 0.0018 (0.05/28, Bonferonni correction); ns indicates P > 0.0018. (DOCX 13 kb) [file 13071_2016_1826_MOESM4_ESM.docx]

Additional file 4: Table S3: Results from a statistical test for linkage disequilibrium for 8 *Lutzomyia vexator* loci. * indicates p<0.0018 (0.05/28, Bonferonni correction); ns indicates p>0.0018.

Lvx9 Lvx67 Lvx123 Lvx179 Lvx504 Lvx7442 Lvx90606 Lvx918

Lvx9 ns ns ns ns ns ns ns

Lvx67 ns ns ns ns ns *

Lvx123 ns * ns ns ns

Lvx179 ns ns * ns

Lvx504 * ns *

Lvx7442 ns *

Lvx90606 ns
